# Validity of new child-specific thoracic gas volume prediction equations for air-displacement plethysmography

**DOI:** 10.1186/1471-2431-6-18

**Published:** 2006-06-05

**Authors:** Paul B Higgins, Analiza M Silva, Luis B Sardinha, Holly R Hull, Michael I Goran, Barbara A Gower, David A Fields

**Affiliations:** 1 Department of Nutrition Sciences, University of Alabama at Birmingham, AL, USA; 2 Exercise and Health Laboratory, Faculty of Human Movement, Technical University of Lisbon, Portugal; 3 Department of Health and Exercise Science, University of Oklahoma, OK, USA; 4 Department of Preventive Medicine, University of Southern California, CA, USA; 5 Department of Pediatrics, Children's Medical Research Institute's Metabolic Research Center, University of Oklahoma Health Science Center, OK, USA; 6 Assistant Professor,University of Oklahoma Health Science Center, School of Medicine,Department of Pediatrics, OUCP Diabetes & Endocrinology, 940 NE 13^th ^Street, CH 2B2426, OKC, OK 73104, USA

## Abstract

**Background:**

To determine the validity of the recently developed child-specific thoracic gas volume (TGV) prediction equations for use in air-displacement plethysmography (ADP) in diverse pediatric populations.

**Methods:**

Three distinct populations were studied: European American and African American children living in Birmingham, Alabama and European children living in Lisbon, Portugal. Each child completed a standard ADP testing protocol, including a measured TGV according to the manufactures software criteria. Measured TGV was compared to the predicted TGV from current adult-based ADP proprietary equations and to the recently developed child-specific TGV equations of Fields *et al*. Similarly, percent body fat, derived using the TGV prediction equations, was compared to percent body fat derived using measured TGV.

**Results:**

Predicted TGV from adult-based equations was significantly different from measured TGV in girls from each of the three ethnic groups (*P *< 0.05), however child-specific TGV estimates did not significantly differ from measured TGV in any of the ethnic or gender groups. Percent body fat estimates using adult-derived and child-specific TGV estimates did not differ significantly from percent body fat measures using measured TGV in any of the groups.

**Conclusion:**

The child-specific TGV equations developed by Fields *et al*. provided a modest improvement over the adult-based TGV equations in an ethnically diverse group of children.

## Background

The consistent increase in the number of children considered overweight or obese in the last thirty years [1] has highlighted the need for an accurate technique to quantify body fat in children. One promising technique is air-displacement plethysmography (ADP), which applies densitometry in determining body composition. A commercially available plethysmographic system (BOD POD^®^) requires minimal testing time and has shown good subject compliance and validity in many populations [2,3].

The BOD POD^® ^consists of a single fiberglass unit comprised of two chambers; a testing chamber and a reference chamber [4]. The subject's body volume is determined based on the pressure-to-volume relationship of the air in the two chambers, as described by Boyle's and Poisson's laws [4]. The testing environment within the BOD POD^® ^operates under adiabatic conditions, *i.e. *air temperature does not remain constant in the testing chamber. As a result of this adiabatic environment, air that maintains a constant temperature, i.e. isothermal-**like **air, in the testing chamber will introduce a significant source of error to the body volume measurement [4-6]. **For example, air in the thoracic cavity will not maintain adiabatic conditions; it will not change its temperature in tandem with the rest of the air in the chamber as it is held close to the temperature of the body, therefore, it is isothermal-like **[4]. Specifically, isothermal and isothermal-**like **air will change its pressure 40% less under compression, relative to adiabatic air. Thus, during an ADP test, in which body volume is determined based on the detection of pressure changes, the presence of isothermal air will result in a lower pressure output signal for a given body volume. Consequently, body volume and ultimately, fat mass will be underestimated [4]. Isothermal-like air is found around the surface of the skin, hair, and in the fabric of clothing. Error from these sources can be minimized by employing standard testing protocols recommended by the manufacturer and others [4,5,7,8]. However, air in the thoracic cavity (lungs and airways) is the major source of **isothermal-like **air and must be taken into account in order to yield accurate estimates of body volume. A measurement of thoracic gas volume (TGV) is obtained during the final portion of the testing protocol and is calculated as the functional residual capacity plus one-half of the tidal lung volume [4].

ADP is ideal for use in pediatric populations, partly because of the short testing duration and the non-invasive nature of the testing protocol [9-15]. However, measurement of TGV requires the subject to breathe through a plastic tube for several cycles of inhalation and exhalation while wearing a nose clip and at the midpoint of exhalation the subject is instructed to puff gently against an occluded valve [4]. This maneuver has proven quite challenging for many populations, but particularly for children. Low compliance rates during TGV measurement have been reported in children, with up to 44% of children unable to obtain a valid measurement in one study [14,16,17]. Typically, when a measured TGV cannot be obtained, TGV is estimated using adult-based equations currently available on the BOD POD^® ^software package [18]. However, when used in children, these proprietary equations have been shown to overestimate TGV and lead to overestimates of fat mass [9,10,14].

In an attempt to improve the accuracy of pediatric TGV prediction, Fields *et al*. developed and validated child-specific TGV equations in a sample of boys and girls between the ages of 6 and 17 years, who were primarily of European American background [16]. To date, one study has attempted to validate these equations in a **European **sample of children [19]. Therefore, the purpose of this study was to validate the Fields *et al*. child-specific TGV equations in children of European, European American, and African American ethnic backgrounds. We found that the child-specific TGV equations developed by Fields *et al*. provided a modest improvement over the adult-based TGV equations.

## Methods

### Birmingham, Alabama sample

The Birmingham sample was comprised of 103 European American and African American children (Table 1). Ethnicity was determined based upon parental and grandparental ethnicity (self-report). For a child to be considered African American, both parents and all four grandparents had to be African American. Children were recruited by advertisements, word of mouth and flyers from the Birmingham metropolitan area. Participants were part of a longitudinal obesity study conducted from 1994 to 2004 of which a sub-sample had their body composition determined by ADP. Subjects arrived at the Department of Nutrition Sciences body composition laboratory for testing after an overnight fast. All children were healthy and free of disorders and medications known to affect body composition and energy metabolism. Approval was obtained from the Institutional Review Board of The University of Alabama at Birmingham prior to testing. Parental consent and child assent were obtained prior to enrollment in the study.

**Table 1:** Subject characteristics by ethnic group and gender

	**European American**	**African American**	**European**
**Girls**	N = 42	N = 23	N = 34

Age (yr)	11.6 ± 1.8	11.1 ± 2.2	14.8 ± 1.7
Height (cm)	152.3 ± 12.3	151.1 ± 13.6	164.2 ± 13.2
Weight (kg)	47.9 ± 13.2	51.1 ± 17.8	55.9 ± 14.6
BMI (kg/m^2^)	20.4 ± 4.4	22.0 ± 6.2	20.2 ± 2.7
**Prevalence of overweight/obesity**^1^	**n = 11**	**n = 10**	**n = 2**

**Boys**	N = 18	N = 20	N = 53

Age (yr)	11.1 ± 1.9	10.2 ± 1.5	15.2 ± 1.4
Height (cm)	151.4 ± 14.6	146.9 ± 12.6	177.3 ± 14.2
Weight (kg)	47.8 ± 16.5	48.1 ± 13.9	69.4 ± 14.7
BMI (kg/m^2^)	20.4 ± 4.7	22.0 ± 5.1	21.9 ± 2.8
**Prevalence of overweight/obesity**^1^	**n = 8**	**n = 8**	**n = 8**

Data are mean ± SD.

^1^Prevalence of overweight/obesity according to international BMI standards in each ethnic-gender group [27].

### Lisbon, Portugal sample

The Lisbon sample was comprised of 87 European boys and girls recruited from five sports clubs in Lisbon, Portugal and were involved in a variety of organized sports teams (*i.e. *swimming, basketball, rugby, gymnastics, and judo) (Table 1). **Subjects trained at least five times a week with each session lasting approximately two hours**. Ethnicity was determined by parental self-report. All children were healthy and free of disorders and medications known to affect body composition and energy metabolism. All subjects were informed about the research design and signed a consent form according to the regulations of the Ethical Committee of the Faculty of Human Movement, Technical University of Lisbon. All testing occurred in the morning after a 12 hour fast.

### ADP testing

Body volume was assessed via air-displacement plethysmography using the BOD POD^® ^software version 1.68 (Life Measurement Incorporated, Concord, CA, USA), at both sites, and according to manufacturer testing recommendations and guidelines. Prior to testing each instrument underwent calibration according to manufacturer guidelines. Each subject wore a swimsuit and cap provided by the respective laboratories and body mass was measured to the nearest 100 g by the ADP system's electronic scale. Details regarding the physical concepts, operational principles, and testing protocol of ADP are described in detail elsewhere [4,20]. After the raw body volume was determined, TGV was measured using the manufacturers previously described protocol and criteria, and was calculated as follows [4]:

TGV = functional residual capacity + (0.5 × tidal volume)

### Prediction of TGV

TGV can be predicted using adult-based proprietary equations available on the BOD POD^® ^software. This prediction is typically used when a measure of TGV cannot be attained. This predicted TGV was determined using the proprietary adult-based equations pre-programmed into the system software and was calculated as follows:

Predicted TGV = estimated functional residual capacity + (0.5 × estimated tidal volume).

The Crapo *et al*. equations are used to estimate functional residual capacity, as described below [18]:

**Men: ***Functional residual capacity (L) = (0.036 × H) +(0.0031 × age) - 3.182*

**Women: ***Functional residual capacity (L) = (0.0472 × H) + (0.009 × age) - 5.290*

Where H is height in centimeters. The Crapo *et al*. functional residual capacity prediction equations were developed from healthy adult male and female subjects aged of 17 to 91 years, using single breath helium dilution techniques [18]. Constants of 1.2 L for males and 0.7 L for females are used to estimate tidal volume and are based on data from adults.

TGV was also predicted using the new child-specific TGV equations of Fields *et al*., developed from direct ADP-derived measures of TGV in a sample of 224 children aged 6 to 17 years. The subject population was comprised of European American, African American, Native American, and Hispanic children making up 87%, 6%, 5%, and 2%, respectively, of the total sample. These equations are detailed below [16]:

**Boys: ***TGV = (0.00056 × H*^2^*) - (0.12422 × H) + 8.15194*

**Girls: ***TGV = (0.00044 × H*^2^*) - (0.09220 × H) + 6.00305*

Where H is height in centimeters. Lastly, final body volume was calculated based upon the initial body volume measurement corrected for TGV (measured and predicted values) and surface area artefact (to account for the isothermal-like air surrounding the surface of the skin). Body density was calculated using this final body volume measurement. Percentage body fat was then determined from body density using the age and gender specific equations of Lohman *et al*. [21]. The technical error of measurement (TEM) and the coefficient of variation (CV) for repeat body volume measurements by the BOD POD^® ^were approximately 0.2 L and 0.5 %, respectively, at both sites.

### Statistical analyses

Differences between the TGV prediction equations, their respective percentage body fats, and measured values were evaluated by ANOVA and Tukey post-hoc tests. R^2 ^and SEE values from regression analyses were used to evaluate the degree of agreement between measured and predicted values. Agreement was further assessed from residual plots using the magnitude of the 95% confidence intervals about the mean differences. Pearson correlation coefficients were also used to determine the extent of bias in the prediction of TGV and percentage body fat across the range of measured values from residual plots. The correlations between measured TGV and (predicted TGV- measured TGV) were used. Statistical significance was set at *P *< 0.05 and data were analyzed using SPSS (version 10; SPSS Inc. Chicago, IL).

## Results

The physical characteristics of the study subjects by gender and ethnicity are in Table 1, a summary of TGV and percent fat values given in Table 2, and summaries of the regression analyses and residual plot analyses are in Tables 3 and 4.

**Table 2:** Thoracic gas volume and percent body fat by group and gender

	**European American**	**African American**	**European**
**Girls**	N = 42	N = 23	N = 34

Measured TGV	2.12 ± 0.55	1.96 ± 0.43	2.79 ± 0.69
Percent body fat (measured TGV)	26.8 ± 9.0	23.5 ± 8.8	18.4 ± 8.1
Predicted TGV by the adult equation **(L)**	2.71 ± 0.42 *	2.67 ± 0.49 **	3.20 ± 0.48**
Percent body fat (predicted adult TGV)	29.3 ± 9.3	26.8 ± 8.8	20.1 ± 8.3
TGV by the child-specific equation **(L)**	2.22 ± 0.46	2.19 ± 0.51	2.83 ± 0.73
Percent body fat (child-specific TGV)	27.1 ± 9.5	24.3 ± 9.1	18.5 ± 8.6

**Boys**	N = 18	N = 20	N = 53

Measured TGV	2.24 ± 0.86	2.09 ± 0.49	4.03 ± 1.00
Percent body fat (measured TGV)	27.2 ± 10.2	25.2 ± 11.2	12.5 ± 5.6
Predicted TGV by the adult equation **(L)**	2.56 ± 0.70	2.34 ± 0.60	3.80 ± 0.66
Percent body fat (predicted adult TGV)	28.6 ± 10.7	26.2 ± 11.0	12.2 ± 5.9
TGV by the child-specific equation **(L)**	2.29 ± 0.75	2.07 ± 0.58	3.82 ± 1.04
Percent body fat (child-specific TGV)	27.4 ± 10.4	25.0 ± 11.0	11.8 ± 5.8

Data are mean ± SD.

Mean differences were assessed using ANOVA and Tukey's test for multiple comparisons.

* *p *< 0.05 and is significantly different from the measured value.

** *p *< 0.001 and is significantly different from the measured value.

**Table 3:** Agreement data for predicted thoracic gas volume

	**Regression**			**Residual Plot**	
	R^2^	SEE	r	*P*	Mean difference (95% CI)

**European American**					
**Girls**					
TGV (adult)	0.56	0.37	-0.65	0.001	0.59 (-0.12;1.30)
TGV (child-specific)	0.61	0.35	-0.56	0.001	0.11 (-0.57;0.78)
**Boys**					
TGV (adult)	0.72	0.47	-0.59	0.01	0.32 (-0.58;1.22)
TGV (child-specific)	0.73	0.46	-0.50	0.05	0.06 (-0.83;0.94)

**African American**					
**Girls**					
TGV (adult)	0.71	0.24	-0.06	0.78	0.71 (0.18;1.24)
TGV (child-specific)	0.73	0.23	-0.01	0.96	0.23 (-0.28;0.75)
**Boys**					
TGV (adult)	0.65	0.29	0.00	0.99	0.26 (-0.44;0.96)
TGV (child-specific)	0.67	0.29	-0.03	0.90	-0.02 (-0.67;0.64)

**European**					
**Girls**					
TGV (adult)	0.64	0.42	-0.72	0.001	0.41 (-0.42;1.23)
TGV (child-specific)	0.76	0.35	-0.16	0.37	0.06 (-0.68;0.75)
**Boys**					
TGV (adult)	0.79	0.46	-0.81	0.001	-0.23 (-1.24;0.78)
TGV (child-specific)	0.80	0.46	-0.16	0.27	-0.22 (-1.15;0.71)

**TGV**; Thoracic gas volume.

**TGV (adult)**; Thoracic gas volume predicted using the adult-based proprietary equations (developed using Crapo *et al*. prediction of functional residual capacity and tidal volume constants for males and females) [18].

**TGV (child-specific)**; Thoracic gas volume predicted using the child-specific equations of Fields *et al*. [16].

**Dependent variable **is the measured TGV.

**r**; The Pearson correlation coefficient between the regression residuals and the criterion; criterion is measured thoracic gas volume).

*P *value indicates whether the r value is significant and estimation bias is present.

**SEE**; Standard error of the estimate.

**Table 4:** Agreement data for percent fat derived using TGV prediction equations

	**Regression**			**Residual Plot**	
	R^2^	SEE	r	*P*	Mean difference(95%CI)

**European American**					
**Girls**					
%FAT(adult)	0.97	1.52	0.07	0.65	2.5 (5.6;0.6)
%FAT (child-specific)	0.98	1.36	0.25	0.11	0.3 (3.3;-2.6)
**Boys**					
%FAT (adult)	0.97	1.84	0.19	0.44	1.4 (5.2;-2.5)
%FAT (child-specific)	0.98	1.60	0.27	0.27	0.1 (3.6;-3.3)

**African American**					
**Girls**					
%FAT (adult)	0.98	1.20	-0.01	0.78	2.8 (5.2;0.5)
%FAT (child-specific)	0.98	1.11	0.19	0.38	0.8 (3.1;-1.5)
**Boys**					
%FAT (adult)	0.99	1.16	-0.19	0.42	1.0 (3.3;-1.2)
%FAT (child-specific)	0.99	1.13	-0.20	0.41	-0.1 (2.1;-2.4)

**European**					
**Girls**					
%FAT (adult)	0.96	1.09	-0.00	0.97	1.7 (5.3;-2.0)
%FAT (child-specific)	0.95	1.24	0.28	0.11	0.1 (2.5;-2.4)
**Boys**					
%FAT (adult)	0.95	1.86	0.22	0.12	-0.7 (3.0;-4.35)
%FAT (child-specific)	0.98	1.16	0.04	0.77	-0.8 (2.4;-4.1)

**%FAT**; percent body fat by air-displacement plethysmography.

**%FAT(adult)**; Percent body fat derived using thoracic gas volume predicted from adult-based proprietary equations (developed using Crapo *et al*. prediction of functional residual capacity and tidal volume constants for males and females) [18].

**%FAT (child-specific)**; Percent body fat derived using thoracic gas volume predicted from child-specific equations by Fields *et al*. [16].

**Dependent variable **is percent body fat derived using measured thoracic gas volume.

**r**; The Pearson correlation coefficient between the regression residuals and the criterion; criterion is percent body fat from measured thoracic gas volume).

*P *value indicates if the r value is significant and hence, the presence of bias.

**SEE**; Standard error of the estimate.

### TGV prediction

TGV predicted from the adult-based equations was significantly higher than measured TGV in all groups of girls (Table 2). However, TGV predicted using the child-specific equations did not differ from measured TGV in the girls (Table 2). In the boys, TGV predicted from both the adult-based and child-specific equations did not differ from measured TGV (Table 2). Child-specific TGV equations performed best in the European boys, explaining 80% of the variance in the measured TGV, with a SEE of 0.46 L (Table 3). The adult-based and child-specific predicted TGV had the poorest agreement in the European American girls explaining only 56% and 61% of the variance in measured TGV, respectively (Table 3). Use of the child-specific equations demonstrated a modest reduction in the magnitude of the 95% confidence intervals about the mean difference relative to the adult-based equations in all groups (Table 3). In the European Americans, significant correlations were observed between the prediction residuals and measured TGV from both the adult-based and child-specific equations. Both sets of prediction equations tended to overestimate TGV at low volumes and underestimate TGV at higher volumes (Table 3). However, no significant correlations were observed in the European children when the child-specific prediction equations were used. In addition, no significant correlations were observed between the prediction residuals and measured TGV from either the adult-based or child-specific TGV equations in African Americans (Table 3).

### Percent body fat prediction

There was no significant difference in percentage body fat derived using either the adult-based or child-specific TGV equations compared to that derived using measured TGV in any of the groups. The percentage fats derived from the adult-based and child-specific TGV equations performed best in African American boys, both explaining 99% of the variance in the percentage fat derived using measured TGV (Table 4). No significant correlations were observed between the predicted percentage fat residuals and percentage fat from measured TGV (Table 4).

## Discussion

This study was conducted to determine the validity of the recently developed child-specific TGV equations in children from three distinct ethnic groups assessed at two different sites. The study sample represented a wide range of age, body mass, and physical activity. We found that the newly developed child-specific TGV equations provided a modest improvement relative to the adult-based TGV prediction equations currently used by ADP software. In addition, we found the equations to be valid for use in African American children. Hence, we recommend the use of these equations for TGV prediction during ADP testing in children of European, European American, and African American background.

Given the recent trends in childhood obesity and its co-morbidities, an accurate and non-invasive technique for body composition assessment is important for research and clinical evaluation [1]. ADP is ideally suited for this assessment because the testing procedure is fast, non-invasive, and has been found to be accurate relative to multi-compartment criterion models in children [12,22]. The initial portion of the testing procedure requires the subject to sit quietly for two periods of 45 seconds each, while raw body volume is determined. The final portion of the testing procedure requires that the subject perform several cycles of balanced tidal breathing through a tube followed by a "puffing" maneuver against an occluded valve. Unfortunately, many children have difficulty meeting the criteria for a valid TGV measurement, which, in all likelihood, is attributable to one or more of the following: 1) unbalanced tidal breathing; 2) an inadequate seal around the breathing tube; or 3) difficulty with performing the "puffing" maneuver. Several investigators have reported difficulty in obtaining an acceptable measure of TGV in children [14,15,22,23]. With substantial technician coaching, the problem can usually be resolved; however, due to time constraints this may not always be possible. In several pediatric studies, investigators did not attempt to measure TGV or were unable to obtain a valid measure [11,13,24-26]. Previously, when unable to obtain a measure of TGV in children, investigators relied on the adult-based TGV prediction equations or in some instances used tedious calculations based on previously published child-specific prediction equations for functional residual capacity and tidal volume [24,25].

At this time, only two studies have assessed the validity of the child-specific TGV prediction equations of Fields *et al*. [19]. Fields *et al*. found the equations to be valid in a group of children similar in age and ethnicity to those in which the equations were developed [16]. Bosy-Westphal *et al*. reported a non-significant overestimation of TGV by the child-specific equations, which represented an improvement over the adult-based equations [19]. Our results are in general agreement with these studies. In the original validation study, Fields *et al*. found the equations to be accurate (R^2 ^= 0.86, SEE = 0.37 L) for TGV prediction in European American boys and girls, similar in age and body composition to the group in which the equations were developed. Agreement was not as strong in the European American children tested in our study. However, the European American children in our sample were younger and had more body fat than those in which the equations were originally developed and validated. Hence, this discrepancy may be related to these age and body composition differences between samples. Bosy-Westphal *et al*. found that the Fields *et al*. equations were more accurate than the adult-based prediction equations in a sample of 224 European boys and girls ranging from five to 18 years [19]. However, the Fields *et al*. equations were found to be similar in accuracy to other equations based on child-specific predictions of functional residual capacity and tidal volume [19]. Nonetheless, the Fields *et al*. equations showed less systematic bias in TGV prediction and are, in general, more user-friendly than the other pediatric prediction equations [19].

Our results indicated the adult-based TGV prediction equations significantly overestimated TGV in each of the three female groups. While these equations also overestimated TGV in the boys, differences were not significant. These findings are similar to those reported in other studies, with discrepancies of 190 to 576 ml between predicted and measured TGV [10,14,16,19]. Mean child-specific predicted TGV did not differ from measured TGV in any of the gender or ethnic groups studied. Although individual agreement among measured and predicted values differed considerably across groups, data from the residual plots revealed a significant improvement in TGV prediction when the child-specific equations were used; biased estimates (determined by Pearson correlation coefficients) were ameliorated in European children and the magnitude of the 95% confidence intervals about the mean differences were reduced in all gender and ethnic groups. However, the overall impact of using either child-specific or adult-based TGV prediction on percent body fat estimation did not differ. Nonetheless, percentage fat derived using the adult-derived TGV equations resulted in an average non-significant overestimation of 2.5 percent fat units in girls and 0.7 percent fat units in boys. Child-specific TGV prediction equations resulted in average overestimates of 0.4 percent fat units in girls, and average under-estimates of 0.2 percent fat units in boys. The 500 ml overestimation of TGV observed when using the adult-based equation in the girls resulted in an average 2% overestimate of percentage body fat. Although, we did not find this statistically different from values derived using the measured TGV, these differences could become significant in larger samples. Hence, the use of the adult-based TGV prediction equations is not recommended in research or clinical settings, particularly in girls.

Our study is the first to assess the validity of the child-specific equations in African American children. A recent study found that adult-based TGV prediction equations were particularly poor in this group [9]. Conversely, error associated with adult-based TGV prediction equations were reported to be small in a sample of African American and European American children. However, the degree of predictive error was assessed in a small sample of children (n = 10), of which the proportion of African American children not reported [15]. Although we found the adult-based equations provided reasonable estimates, slight improvements in individual percent body fat estimation using TGV prediction equations were made when the child-specific TGV equations were applied. Hence, we also recommend the use of the Fields *et al*. equations when measures of TGV cannot be attained in this group.

Of note we found the child-specific TGV equations to be valid in a group of **very active European children**. Unfortunately, we did not have good estimates of time spent in sports and activity in any of the subjects, and therefore we cannot assume that the child-specific equations are valid in athletic children. However, given all children in the European sample were participating in a variety of sports, our results suggest that the child-specific equations may also be appropriate for use in athletic children. Obviously, this will require further verification in studies with adequate measures of physical activity.

## Conclusion

In summary, we found the new child-specific TGV equations to be valid for use in children across a range of age, gender, and ethnicity. The equations offered significant improvement over the current adult-based prediction equations in girls. Improvement over adult-based prediction was more modest in the boys. In conclusion, when possible, TGV should be measured according to software criteria. However, if a successful measurement cannot be attained, we recommend use of the newly developed child-specific TGV equations in children aged 5 to 17 years.

## Competing interests

David A. Fields has received funding from Life Measurement Incorporated for past studies (though Life Measurement Incorporated did not fund this study). None of the authors has financial or other interests or own stock or are applying for patents that may represent a conflict of interest.

## Authors' contributions

PBH drafted the manuscript, performed statistical analysis, interpreted, and evaluated the data. AMS drafted the manuscript, performed statistical analysis, and carried out ADP testing on the Portugal cohort. LBS provided critical evaluation on ADP testing on the Portugal cohort and drafted the manuscript. HRH provided critical review of the manuscript and participated in writing of the manuscript. MIG oversaw the collection of ADP testing in the Birmingham cohort and provided critical review of the statistical analysis and the manuscript. BAG oversaw the collection of ADP testing in the Birmingham cohort and provided critical review of the statistical analysis and the manuscript. DAF conceived and drafted the manuscript.

## Pre-publication history

The pre-publication history for this paper can be accessed here:


